# Comparison of US Strain Elastography and Entero-MRI to Typify the Mesenteric and Bowel Wall Changes during Crohn's Disease: A Pilot Study

**DOI:** 10.1155/2017/4257987

**Published:** 2017-10-30

**Authors:** Giuseppe Lo Re, Dario Picone, Federica Vernuccio, Laura Scopelliti, Ambra Di Piazza, Chiara Tudisca, Salvatore Serraino, Giambattista Privitera, Federico Midiri, Sergio Salerno, Massimo Midiri, Tommaso Vincenzo Bartolotta, Roberto Lagalla

**Affiliations:** ^1^Section of Radiology-Di.Bi.Med., University of Palermo, Palermo, Italy; ^2^AOU Policlinico-Vittorio Emanuele, UOC Radiodiagnostica Ospedale Vittorio Emanuele, Catania, Italy

## Abstract

**Purpose:**

To evaluate and compare the mesenteric and bowel wall changes during Crohn's disease (CD) on ultrasonography (US) Strain Elastography (SE) and Enterography Magnetic Resonance Imaging (E-MRI).

**Methods:**

From July 2014 to September 2016, 35 patients with ileocolonoscopy diagnosis of CD were prospectively examined with E-MRI and in the same time with US and SE.

**Results:**

A total of 41 affected bowel segments and 35 unaffected bowel segments in 35 patients were evaluated. US-SE color-scale coding showed a blue color pattern in the fibrotic mesentery and bowel wall in 15 patients and a green color pattern in the edematous ones in 20 patients. The signal of the bowel wall and mesenteric fat was iso/hypointense on T2-weighted sequence in the fibrotic pattern (23/35 and 12/35 patients) and hyperintense in the edematous pattern (12/35 and 23/35 patients). Mean ADC values were, respectively, 2,58 ± 0,33 × 10^−3^ for the fibrotic mesentery and 2,14 ± 0,28 × 10^−3^ for edematous one. There was a statistical correlation between US-SE color-scale and T2 signal intensity and between the US-SE color-scale and ADC maps.

**Conclusions:**

US-SE, ADC, and signal intensity on T2-weighted sequences on MR prove to be useful tools for the evaluation of CD pattern.

## 1. Introduction

Crohn's disease (CD) is relapsing and remitting form of transmural inflammatory bowel disease that affects the gastrointestinal and diffuses into the mesenteric fat [1]. The incidence and prevalence are increasing with time and in many regions around the world with highest reported prevalence in Europe and North America [2].

Although endoscopy with biopsy remains the gold standard for CD's diagnosis, imaging plays a central role in the assessment of intestinal and extraintestinal involvement, in the surveillance and assessment of response to treatment, and in the detection of complications [1]. Many imaging tools help in identifying bowel wall thickening, hyperperfusion, and active inflammation, as Enterography Magnetic Resonance Imaging (E-MRI) and Contrast-Enhanced Ultrasonography [3–5]. On the contrary, the distinction between inflammation and fibrosis may still be tricky. However, this differentiation has a great impact on patients' management: fibrotic pattern usually needs endoscopic dilatation or surgery, while active inflammation pattern responds to the conservatory treatment [1].

The evaluation of elasticity at ultrasonography (US), through Strain Elastography (SE), is a noninvasive method that allows assessing tissue stiffness, and it is already used for the evaluation of liver, breast, and thyroid diseases [6]. SE is able to quantify tissue elasticity simultaneously during B-mode ultrasound examination and elasticity values can be on the basis of anatomical information. SE allows seeing real-time visualization of an image on the display screen and the stress is applied manually via a hand-held ultrasound transducer, which provides low-frequency compression. In particular, SE provides elastography colorimetric maps according to the degree of stiffness.

Recent clinical studies reported promising results for distinguishing inflamed from fibrotic bowel by using the US elastography [7–10]. The overwhelming majority of investigations evaluating the radiological assessment of inflammatory bowel disease lack an appropriate study of the mesenteric fat. To our knowledge, SE of the mesenteric fat has not been studied in in vivo human CD and in particular two SE and E-MRI techniques have not been compared yet for the differentiation between edematous and fibrotic changes in CD.

The purpose of our study was to assess whether SE and DWI could be used detect mesenteric and bowel wall fibrosis and differentiate it from edematous/inflammatory changes.

## 2. Materials and Methods

### 2.1. Patients

The institutional review board approved this prospective study. All patients gave written informed consent. From July 2014 to September 2016, 35 patients (mean age 33,12; range 9–90; 16/19 F/M) were selected for this study, and all patients had a previous ileocolonoscopy diagnosis of CD. Patients were categorized according to the Montreal Classification [11]. Demographic data of the patients were collected.

Typical E-MR imaging appearance of CD was defined as bowel wall thickening (>1 cm) and bowel wall enhancement, while the extramural disease was defined as fibrofatty proliferation as thickening of extramural fat, which separates bowel loops and the vascular engorgement (comb sign) [1].

Typical US imaging appearance of CD was defined as bowel wall thickening (>3 mm), losing or preserving the gut signature, while the extramural disease was defined as perienteric fluid and creeping fat which separates bowel loops [1].

### 2.2. MR Imaging Protocol

All 35 patients were imaged with a 1.5T MR imaging unit (Achieva, Philips Healthcare, Best, Netherlands). A dedicated abdominal multichannel surface coil was used for all patients. All patients were scanned after oral administration of 1.5–2 L of polyethylene glycol solution, Moviprep (Norgine, Amsterdam, The Netherlands), or Selg-Esse 1000 (PROMEFARM, Milan, Italy), as oral contrast agent over the course of 30 minutes. Imaging protocol included axial precontrast images acquired with T2-weighted fast-spin echo sequence (TR/TE, 4000/76 ms; section thickness 5-6 mm) and T1-weighted axial in-phase and out-of-phase gradient-recalled-echo sequence (TR/TE, 140/2.2–4.4 ms; section thickness, 5-6 mm). Dynamic studies were performed with three-dimensional fat- suppressed T1-weighted gradient echo sequence (TR/TE, 3.8/1.2 ms; FA 12°; slice thickness: 4.4 mm; intersection gap 2 mm; FOV: 44 cm; matrix 256 × 256) by using a bolus tracking system. Images were acquired on the coronal plane immediately before and after intravenous injection of either 0.1 mmol/kg body weight of gadoterate meglumine (Dotarem, Guerbet GmbH, Sulzbach, Germany) at 2 mL/s or 0.1 mmol//kg body weight through a 20-gauge intravenous catheter by means of a power injector (Medrad Spectris Solaris EP MR Injection System; Bayer Healthcare), followed by a 20-mL saline flush at the same injection rate. Scanning delays after automatic detection of contrast bolus were 18, 60, and 180 s, respectively for the acquisition of the arterial, portal venous, and three-minute phase. Before contrast-medium injection, 20 mg hyoscine-N-butylbromide (Buscopan, Boehringer, Ingelheim, Germany) was intravenously injected unless contraindicated (e.g., history of cardiac arrhythmia, narrow angle glaucoma, or prostatism). Diffusion-weighted (DWI) single-shot echo planar images with *b* values of 0, 600, and 800 s/mm2 were acquired with free breathing method using slice thickness of 8 mm.

### 2.3. US-SE Imaging Protocol

All US examinations were conducted by (blinded) abdominal staff radiologist with 15 years of experience and another (blinded), in his fifth year of training, respectively, by using a Samsung RS80A with Prestige (Samsung Medison Co. Ltd) that included the EZU-TE5 option. All patients underwent abdominal US examination soon after the E-MR. B-mode US was conducted with a linear-array transducer (EUP-L74M, 5–13 MHz, 50 3 10 mm; Samsung) equipped with ElastoScan technology.

For each patient, B-mode US coronal and axial images were obtained of the last ileal loop and of the relative perimesenteric fat. In the same locations, the US-SE was performed according to manufacturer guidelines with the same transducer, and relative strain measurements were obtained from the perimesenteric fat.

For every patient, B-mode US coronal and axial images were obtained of the ileal loop and of the relative perimesenteric fat in the upper-left abdominal location. In the same locations, the US-SE was performed according to manufacturer guidelines with the same transducer, and relative strain measurements were obtained from the perimesenteric fat.

### 2.4. Image Analysis

All MR images were evaluated on a commercial picture archiving and communication system station (PACS-Impax, Agfa-Gevaert). An acute inflammatory change of CD appearance was defined as a bowel wall thickening greater than 3 mm, hyperintense bowel wall on T2 FAT-SAT-weighted images and increased enhancement on postcontrastographic T1-weighted images as stratified contrast enhancement pattern or transmural enhancement pattern, while the mesenteric is characterized by edema as an intermediate to high signal intensity on T2 FAT-SAT-weighted images and by the presence of inflammatory mesenteric lymph nodes.

While a fibrotic change of CD appearance was defined as a bowel wall thickening greater than 3 mm, hypointense bowel wall on T2 FAT-SAT-weighted images and increased enhancement on postcontrastographic T1-weighted images were defined as low level inhomogeneous contrast enhancement pattern. The mesenteric is characterized by creeping fat as an increased mesenteric fat producing a mass effect which presented a slightly decreased signal intensity on T2 FAT-SAT-weighted images [1]. Two observers (blinded), an abdominal radiologist with 15 years of experience and another, in his fifth year of training, graded jointly their degree of confidence in diagnosing the presence of an acute inflammatory or fibrotic appearances in volumetric T1 weighted GRE images on postcontrastographic phases, in T2 FAT-SAT-weighted images and DWI- weighted images (1: no changes; 2: probably acute inflammatory; 3: definitely acute inflammatory; 4: probably fibrotic; 5: definitely fibrotic). If there was any discordance, the determination was reached by means of consensus. Disagreements among the three readers were considered relatively few and minor. Observers also analyzed the number and diameter of bowel wall lesions and the presence of any complications.

Observers analyzed the US-SE of the patients and they categorized jointly their degree of confidence in diagnosing the presence of an acute inflammatory or fibrotic appearances of the mesenteric fat in colorimetric map images (red: no changes; yellow: probably acute inflammatory; green: definitely acute inflammatory; light blue: probably fibrotic; blue: definitely fibrotic); we followed the methods of Dioguardi Burgio et al. 2016 [12].

### 2.5. Statistical Analysis

Values are expressed as mean (range) or percentages, as appropriate. The Fisher exact test was used to compare differences in discrete or categorical variables and Student's *t*-test was used to compare differences between mean values. Statistical significance was defined as *p* less than 0.05. All statistical tests were 2-sided. All statistical analyses were performed by using GraphPad Prism for Macintosh version 6 software (GraphPad Software Inc., La Jolla, CA, USA). Graphs were created by using the same software.

## 3. Results

Affected and unaffected mesenteric areas and bowel walls were detected on E-MRI and US-SE. A total of 41 affected bowel segments (35 ileum and 6 colon segments) and 35 unaffected bowel segments (35 ileum) in 35 patients were evaluated. In 29 patients the ileum was affected while in 6 patients the ileum and colon were involved. The affected segment's extension mean was 8,1 cm (range 2,1 cm–25,2 cm) and the wall thickening mean was 1 cm (range 0,6 cm–1,3 cm). We found 9 stricture segments while intrawall abscess and ileoileal fistulas were detected in 11 and 7 patients, respectively.

**Figure 1:**
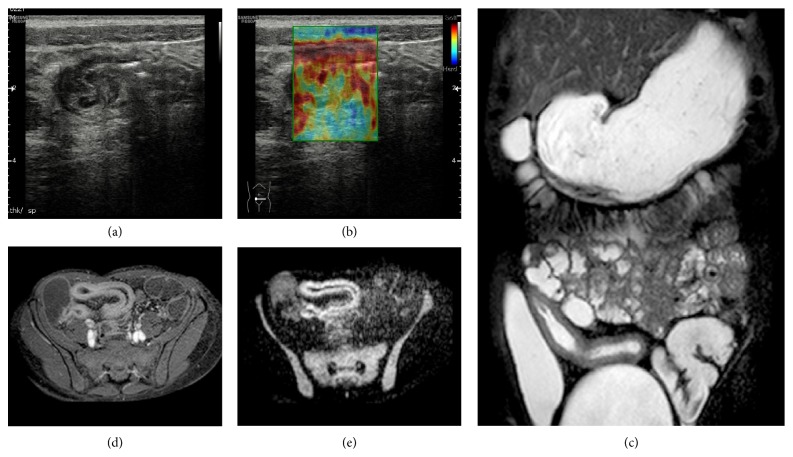
Longitudinal US-scan of the last ileal loop in a 33-year-old male shows wall thickening with prominence of submucosa and hyperechoic appearance of perivisceral fat. The gray scale (a) findings and US-SE (b) aspect performed at this level are suggestive for active inflammation pattern. The MR study ((c), (d), and (e)) confirmed these suspicions: T2 weighted coronal view revealed hyperintensity of the bowel wall, T1 weighted axial postcontrast showed stratification of the bowel wall, DWI with 800 *b* value showed hyperintensity of the bowel wall, and an aspect of the last ileal loop is very similar with US findings.

**Figure 2:**
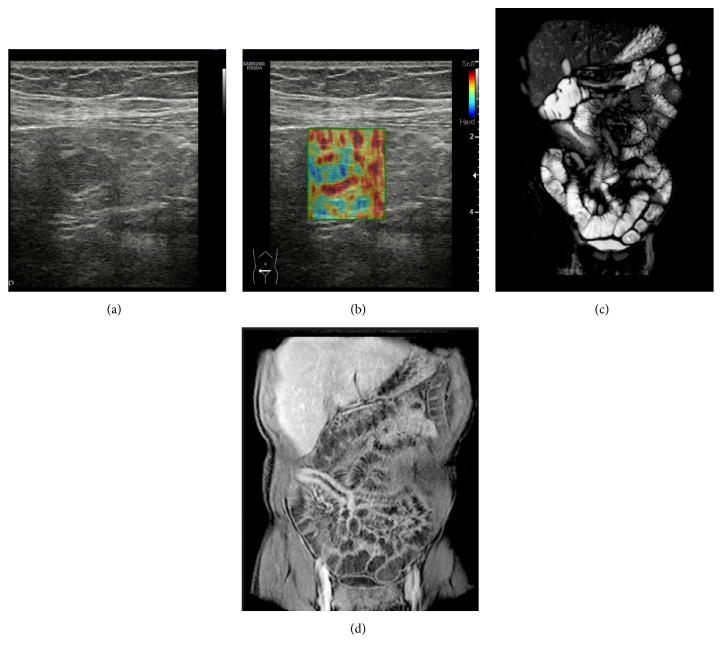
Transverse US-scan of the last ileal loop in a 26-year-old female shows wall thickening in which stratification was completely lost and there is hypoechoic appearance of perivisceral fat. The gray scale (a) findings and US-SE (b) aspect performed at this level are suggestive for fibrotic pattern. The MR study ((c) and (d)) confirmed these suspicions: T2 weighted coronal view revealed hypointensity of the bowel wall, T1 weighted coronal postcontrast showed enhancing of the bowel wall without stratification.

A fibrotic mesenteric and bowel wall change were present on E-MRI and US-SE in 12/35 (34,29%), 23/35 (65,71%), 15/35 (42,85%), and 20/35 (57,15%), respectively, while an acute inflammatory mesenteric and bowel wall change were present on E-MRI and US-SE in 23/35 (65,71%), 12/35 (34,29%), 20/35 (57,15%), and 15/35 (42,85%), respectively (Figures 1 and 2).

In all unaffected bowel segments and relative perimesenteric fat, the US-SE color- scale coding showed a red and/or yellow color variation. Moreover, the signal of the bowel wall and mesenteric fat were iso/hypointense on T2-weighted sequence in the fibrotic pattern (23/35 and 12/35 patients) and hyperintense in the edematous pattern (12/35 and 23/35 patients). The correlation between US-SE color-scale and T2 signal intensity was statistically significant (*p* < 0,05).

There was a significant diffusion restriction in 18 patients with CD in the active phase (mean ADC values for the fibrotic mesentery: 2,58 ± 0,33 × 10^−3^, mean ADC values for edematous mesentery: 2,14 ± 0,28 × 10^−3^). There was a statistical correlation between the US-SE color-scale and ADC maps (*p* < 0,05). However, 3/35 patients were defined as acute inflammatory pattern by US-SE but E-MRI through both T2-weighted sequence and ADC map classified them in fibrotic pattern.

No significant diffusion restriction was encountered in 35/35 unaffected bowel segments and relative perimesenteric fat (mean ADC values: 2,92 ± 0,15 × 10^−3^). Also we found a statically significant correlation between the presence of enlarged lymph nodes and the edematous change (*p* < 0,05) and between the presence of stricture and the fibrotic changes (*p* < 0,05).

However, there was a significant difference between the control and the study group. According to the data in the control group there was not a significant diffusion restriction and there were an isointense T2 signal and a predominantly green USE color-scale of the mesentery and bowel wall.

## 4. Discussion

In the management of CD, key important tools to guide therapeutic choices are the assessment of disease activity and the differentiation between inflammatory/edematous and fibrotic patterns [1]. MRI examination through DWI helps in the detection of active CD, showing high signal on DWI sequences, and in the differentiation between fibrotic and edematous changes of bowel loops, showing higher ADC values in case of fibrotic changes compared to the edematous mesentery, and of this latter one compared to the normal pattern.

The main guiding tool for patient management is the differentiation between active edematous inflammation, which needs just conservatory treatment, and progression towards fibrosis, which usually needs endoscopic dilatation or surgery [7]. Thus, the main goal of our study was to evaluate the role of US/SE itself and in comparison to E-MRI in the differentiation of fibrotic and edematous pattern of CD. Our results show that, on E-MRI, fibrotic changes of the mesentery are characterized by higher ADC values and lower T2 signal compared to the edematous mesentery.

MRI is useful in the differentiation between active inflammation and fibrostenotic disease through the evaluation of mural thickness, stratification and enhancement, mural edema, mesenteric vascularity/adenopathy, fibrofatty proliferation, and the evaluation of the presence of complications. Rimola et al. classified the different stages of inflammation and proved that MRI may accurately detect severe fibrosis through the evaluation of the enhancement pattern of affected bowel loop [13]. In a meta-analysis of seven studies (140 patients) [14], MRI correctly graded disease activity in 91% of cases with frank disease.

However, MRI is time and money consuming and not widely available. US, which is more widely available and less time consuming than MRI, may evaluate the localization and the length of the affected intestinal segments in CD (bowel thickening from the superficial layer which is hyperechoic to the mucosal layer > 5 mm in a nondistended bowel, >3 mm in a distended bowel; loss of multilayer pattern is sign of active inflammation) [5]. Concerning the mesenteric changes, it may assess the presence of creeping fat as an echogenic area separating bowel loops representing the fibrofatty proliferation of adipose tissue that extends around active inflammation. Our results proved that fibrotic and edematous changes of the bowel and mesentery in CD may be done noninvasively through SE, which is a noninvasive method for evaluating tissue stiffness. SE has initially been used to assess breast, thyroid, and liver lesions, but in the last decade this method was found to have many applications in a wide range of fields [6, 15, 16].

At elastography red means soft while blue means hard: so the nearer you are to the blue the nearer you are to the fibrosis while the nearer you are to the red the nearer you are to the normal pattern. Hence, it is possible to grade the fibrofatty changes of the mesentery from normal (grades 1 and 2) throughout grade 3 (green) edematous changes to blue (grades 4 and 5) which indicates fibrotic changes [17]. In our study, concerning the changes in the stiffness at SE, the pathological ileal loop showed edematous changes (grade 3, green) in 20 patients and fibrotic changes in 15 patients (grades 4 and 5, light and dark blue), while all the nonpathological ileal lops did not show any oedematous or fibrotic change (grades 1 and 2, red and yellow color). These differences proved to be statistically significant in the identification of the pathological loop and in the differentiation of the fibrotic and edematous changes. A close look at our data shows that in 3 patients US-SE did not correctly grade as fibrotic the bowel and mesenteric changes. This could be explained by the fact that a qualitative assessment of US-SE leads to incomplete separation of edematous and low-fibrosis pattern and of high- and low-fibrosis pattern, while on MRI the combination of the quantitative datum of ADC map and the T2 signal allows a more precise categorization. Although this overlap is minimal, a quantitative assessment through shear wave US elastography could theoretically overcome this limit.

Few studies in the literature investigated the possibility of evaluating the bowel by contrast-enhanced ultrasonography by use of oral (SICUS) or intravenous (CEUS) contrast agents. SICUS has emerged as a valuable and radiation-free technique [18] in the evaluation of bowel wall in CD while the use of CEUS is important to monitor response to therapies [19, 20].

Few studies in the literature investigated the possibility of evaluating the bowel by US- elastography [7–10, 17], but, to date, this imaging technique has not been introduced in the common diagnostic strategies of CD patients yet [5]. Dillman et al. demonstrated that shear wave imaging accurately distinguishes fibrotic bowel in a CD animal model [8] and in ex vivo bowel wall loops [7].

Our study revealed a statistically significant correlation between the MRI and the US-SE findings during the evaluation of bowel wall and mesenteric changes. In particular, the bowel wall thickness and stratification pattern can be seen on US and the additional SE score gives information on the associated mesenteric changes. The mesenteric white adipose tissue, which was long considered as the “anatomists' Cinderella,” is now recognized as a multifunctional organ. The fat abnormalities in CD correlate with transmural inflammation, with a significant relationship between fat wrapping and other connective tissue changes, including fibrosis, muscular hypertrophy, and stricture formation. As demonstrated by our study, the evaluation of the mesenteric changes in CD through US-SE can be considered an additional tool in the differentiation of the edematous or fibrotic changes, in order to distinguish acutely inflamed from fibrotic intestine in patients with CD. Increased ADC values and blue color in inflammatory stricture bowel is likely due to differences in bowel wall collagen and water content compared to edematous bowel wall. US strain imaging quantifies the “hardness” or “softness” of a tissue as a function of tissue compressibility. This differentiation is mandatory for the therapeutic management. A strength of our evaluation is surely the performance of SE soon after E-MRI, and thus the effect of the spasmolytic agent may have helped in the evaluation of the bowel wall and mesentery stiffness. Many limits pertain to our analysis. Firstly, we performed just a qualitative evaluation of edematous or fibrotic changes at SE. Actually, quantitative and comparative analyses may be performed through newly available software programs and these could better demonstrate the correlation between MRI and SE findings. Moreover, considering that there is minimal overlap between edematous and fibrotic pattern, quantitative method could turn to be useful in the differentiation and could help in identifying a possible cut-off value in KPascal between affected and unaffected mesentery and between fibrotic and edematous mesentery. Another limitation of our study is the small number of patients enrolled and thus the small number of bowel wall loops evaluated and a low number of patients for an accurate comparison between elastography and MR. Finally, intrinsic limitations pertain to US. For example, obesity in patients with CD might impair the applicability of SE in these patients by causing inadequate penetration of the high-frequency transducers.

## Abbreviations

CD: Crohn's disease
US: Ultrasonography
SE: Strain Elastography
DWI: Diffusion-weighted imaging
ADC: Apparent diffusion coefficient
E-MRI: Enterography Magnetic Resonance Imaging.
